# Competing risk models to evaluate the factors for time to loss to follow-up among tuberculosis patients at Ambo General Hospital

**DOI:** 10.1186/s13690-023-01130-2

**Published:** 2023-06-25

**Authors:** Daba Bulto Fufa, Tadele Akeba Diriba, Kenenisa Tadesse Dame, Legesse Kassa Debusho

**Affiliations:** 1 Department of Statistics, College of Natural Sciences, Jimma University, Jimma, Ethiopia; 2 Current address: Department of Statistics, Assosa University, Assosa, Ethiopia; 3 Department of Statistics, College of Science, Engineering and Technology, University of South Africa, Christian de Wet Road and Pioneer Avenue, Private Bag X6 Florida, 1710 Johannesburg, South Africa

**Keywords:** Competing risks, Loss-to-follow-up, Hazard functions, Tuberculosis

## Abstract

**Background:**

A major challenge for most tuberculosis programs is the inability of tuberculosis patients to complete treatment for one reason or another. Failure to complete the treatment contributes to the emergence of multidrug-resistant TB. This study aimed to evaluate the risk factors for time to loss to follow-up treatment by considering death as a competing risk event among tuberculosis patients admitted to directly observed treatment short course at Ambo General Hospital, Ambo, Ethiopia.

**Methods:**

Data collected from 457 tuberculosis patients from January 2018 to January 2022 were used for the analysis. The cause-specific hazard and sub-distribution hazard models for competing risks were used to model the outcome of interest and to identify the prognostic factors associated to treatment loss to follow-up. Loss to follow-up was used as an outcome measure and death as a competing event.

**Results:**

Of the 457 tuberculosis patients enrolled, 54 (11.8%) were loss to follow-up their treatment and 33 (7.2%) died during the follow up period. The median time of loss to follow-up starting from the date of treatment initiation was 4.2 months. The cause-specific hazard and sub-distribution hazard models revealed that sex, place of residence, HIV status, contact history, age and baseline weights of patients were significant risk factors associated with time to loss to follow-up treatment. The findings showed that the estimates of the covariates effects were different for the cause specific and sub-distribution hazard models. The maximum relative difference observed for the covariate between the cause specific and sub-distribution hazard ratios was 12.2%.

**Conclusions:**

Patients who were male, rural residents, HIV positive, and aged 41 years or older were at higher risk of loss to follow-up their treatment. This underlines the need that tuberculosis patients, especially those in risk categories, be made aware of the length of the directly observed treatment short course and the effects of discontinuing treatment.

**Supplementary Information:**

The online version contains supplementary material available at 10.1186/s13690-023-01130-2.

**Table:** 

Text box 1. Contributions to the literature	
• Research has shown that the competing risk models emphasis on various measures, which may result in outcomes and interpretations that varied greatly.	
• This study contributes to recognized gaps in the literature, including ascertaining which competing risk models are suitable for identifying associations or risk sets in survival analysis by considering the construction of risk sets and interpretation of the underlying hazard function.	
• Findings from this study may help researchers in identifying the proper application of the methods and the correct interpretation of the acquired data by quantifying changes in the hazard models, and the knowledge may be helpful in making policy decisions.	

## Background and motivation

Tuberculosis (TB) is a chronic respiratory infectious disease, and one of the major public health problems worldwide [1–3]. Even though several treatment strategies are available to manage this disease, TB remains a leading cause of death globally [2]. According to the WHO Global TB report, an estimated 10 million TB cases and 1.2 million deaths were recorded in 2019 [4]. The African continent shared an estimated 25% of the global TB cases [4]. Of the 1.5 million tuberculosis deaths in 2020, 214,000 reported deaths were among people living with HIV and co-infected with TB [2]. The increase in TB deaths has occurred mainly in thirty countries with the highest TB-burden, sixteen of which are in Africa [2]. Ethiopia, an East African country, was ranked tenth among these countries with an estimated TB incidence of 140 cases per 100,000 population [4]. Hospital statistical data from the Ethiopian Federal Ministry of Health (MOH) shows that tuberculosis is the leading cause of morbidity and the second leading cause of death after malaria [5].

To reduce mortality and prevent TB transmission, it is important to follow various TB treatment strategies. A major challenge facing most TB programs occurred when TB patients are unable to complete their treatment for one reason or another [6]. In 2013, the WHO decided to use the term “loss to follow-up (LTFU)” for TB patients who have not started treatment or whose treatment has been interrupted for more than two consecutive months [7]. Failure to complete the treatment contributes to the emergence of multi-drug resistant TB, making these patients more likely to develop infectious active TB again [8, 9]. Ethiopia has been implementing the directly observed therapy short course (DOTS) since 1991. For DOTS-registered TB patients, observing alone while on medication is not sufficient to prevent LTFU treatment. Therefore, understanding the survival time from treatment initiation to LTFU in TB patients and assessing risk factors for LTFU treatment are essential for designing time relevant intervention strategies.

In survival analysis, various methods are available to examine data sets defined in terms of the time from a well-defined time origin to the occurrence of a particular event [10]. The determinants affecting time to treatment LTFU can be examined by considering death event as a censored observation in survival analysis. However, TB patients admitted to the DOTS program are followed for 6 months and death is considered a competing risk event whenever LTFU is the primary event. In this situation, standard survival methods are inadequate to analyze survival data in a competing risk setting [11]. Death from any cause in TB patients precludes observation of LTFU treatment. A major limitation of modeling time-to-event data in the presence of competing events is that when estimating regression parameters under a specific cause, individuals failing from causes other than the cause of interest are considered censored observations. For instance, Larson and Dinse [12] proposed to use logistic and piece-wise exponential regression models to assess the influence of the covariates on the event of interest and the covariates effects on failure time given the type of event, respectively. A more flexible approach to survival time was also introduced using a generalized three-parameter gamma distribution and a generalized estimating equation [13, 14].

One of the most popular methods of analyzing the competing risk data is the Cox proportional hazard (CPH) model. The CPH is used to examine the effect of covariates on the cause-specific hazard (CSH) function [10]. Furthermore, since the Kaplan-Meier (KM) method is not suitable for analyzing patient survival when there are competing risks, Fine and Gray [15] designed a new approach based on the cumulative incidence function (CIF). The CIF describes the probability of an event occurring before a certain point in time. In contrast to the CPH model, the CIF-based model do not ignore other competing risks when a particular cause is of interest, and their estimates are interdependent [16]. Moreover, unlike the KM method, competing events are not handled as regular censoring events and are computed without influence on the CIF for the event of interest [17].

Statistically analyzing and inferring competing risks data is not straightforward due to the variety of measurement techniques available in regression modeling and the variety of methodological approaches to analyzing time-to-event data in the presence of competing events. Many sources of error can occur in situations where mutually exclusive event types are available. Therefore, in this study, the authors focused on modeling time to LTFU treatment among TB patients using competing risk models. To the best of the authors’ knowledge, no similar work has been done using competing risk models to analyze TB in Ethiopia.

The rest of the paper is organized as follows. The data, some basic review of competing risk models for analyzing survival data, regression models for CSH and sub-distribution hazard (SDH) and inferences related them are introduced in “Methodology” section. The results from applying these methods on the study data are discussed in “Results” section. Finally, the “Discussion” and “Conclusion” sections, respectively, provide the discussion and conclusions, along with recommendations for further investigation.

## Methodology

### Study area

The data for this study was obtained from Ambo General Hospital (AGH). AGH is located in the city of Ambo, West Showa zone of the Oromia regional state in Ethiopia. Ambo is 114 km away from the capital city, Addis Ababa, to the west between latitude $8^{\circ }$56’30” - $8^{\circ }$59’30” North and longitude $37^{\circ }$47’30” - $37^{\circ }$55’15” East.

### Sampling and data collection procedure

In Ethiopia a national TB program follows the DOTS strategy and uses standard international criteria for the diagnosis and treatment of TB patients [18]. A retrospective cohort study was conducted to assess time to LTFU TB treatment. The data used to identify risk factors for LTFU were extracted from the registration log book and patients’ registration cards. The population for this study was all TB patients enrolled at AGH for treatment between January $1^{st}$, 2018, and January $30^{th}$, 2022. All the data was carefully reviewed from the registration log book and patients’ registration cards. Any counters with insufficient information were removed from the file and excluded from the analysis.

### Diagnosis and treatment of pulmonary and extra pulmonary TB in Ethiopia

A national TB program in Ethiopia adopts accepted worldwide standards for the diagnosis and treatment of TB patients and adheres to the DOTS strategy [7]. The diagnosis of pulmonary TB is established in accordance with WHO standards [18]. A patient is considered to have Smear-positive pulmonary TB (SPPTB) if he/she has symptoms or signs suggestive of pulmonary TB (involving the lung parenchyma) and whose smear microscopy is positive (AFB positive). Smear-negative pulmonary TB (SNPTB) is defined as a patient with pulmonary TB symptoms or signs who tested negative on a smear microscopic examination, but who has been confirmed as having active TB by a skilled medical professional and decided to be given a full course of TB treatment. Whereas, a patient is deemed to have extra pulmonary TB (EPTB) if he/she has any TB cases that have been bacteriology confirmed or histologically or clinically diagnosed case of TB involving organs other than the lungs, such as lymph nodes, pleura, abdomen, genitourinary tract, joints and bones, meninges.

All instances of pulmonary and EPTB are handled with the same first-line anti-tuberculosis standard treatment. Patients are given rifampicin, pyrazinamide, isoniazid, and ethambutol daily for the first two months (the initial phase), then rifampicin and isoniazid daily for the following four months (the continuation phase). Sputum microscopy examination of pulmonary TB patients is carried out during follow-up, however those with EPTB are clinically monitored, and in particular, body weight is measured and recorded during treatment on personal treatment cards (at two, five, and six months).

### Inclusion and exclusion criteria

All patients who received TB treatment at AGH during the study period and who registered full information on their registration log book or patient identification cards were eligible for the study. Patients who have not started treatment for TB and who do not have sufficient information about one of the vital variables either in the registration book or on the card were not eligible. In addition, patients who returned to hospital within the study period, patients on anti-TB drugs previously and LTFU with positive or negative bacteriology, and patients who experienced disease at any anatomical site were excluded from the study.

### Variables

#### Dependent variables

In this study, the response variable was the time (in months) to LTFU treatment among TB patients starting from the months the patients registered at hospital. The status of the patient is 1 if interested event occurred (LTFU), 2 if death occurred due to any causes in TB patients (competing risk event), and 0 for events censored. The survival time was defined as the time in months from the beginning of TB treatment to LTFU as the main or associated cause. Death due to any cause in TB patients under follow-up was considered as competing event. Censoring occurred at either the end of the study or complete treatment.

#### Independent variables

The explanatory variables included were sex, residence (Urban and Rural), patient category (New, Relapse, Failure, Transfer in), Weight loss (No, Yes), Types of TB (Extra pulmonary tuberculosis (EPTB), Smear positive pulmonary tuberculosis (SPPTB), Smear negative pulmonary tuberculosis (SNPTB)), HIV status (Negative, Positive), contact history (No, Yes), age in years (0-18, 19-40, 41-60, > 60), baseline weight in kg (< 20, 20-29, 30-37, 38-54, >55).

### Statistical analyses

In this study, competing risk models were employed to identify factors for time to LTFU treatment among TB patients at AGH. Under the frame work of competing risks modeling the two most commonly used approaches are the CSH Cox approach and Fine-Gray proportional SDH model [19]. In the standard survival analysis, Cox proportional hazards model is a semi-parametric model in which dependence on the explanatory variables is modelled explicitly but no specific probability distribution is assumed for survival times. An analogous Cox regression approach can also be applied using CSHs regression when competing risks are present. In the analysis of times to a certain event *k*, the CSH is the instantaneous rate of experiencing cause *k* amongst those who are event-free (i.e., have not yet had cause *k* or any of the competing events). A straightforward way of applying this CSH approach is to fit a separate Cox model for each cause, censoring any competing events at their time of occurrence.

The one-to-one correspondence between hazard and survival that exists in the standard survival analysis does not necessarily hold when competing risks are present [20]. As a consequence, the effect of a covariate on the CSH for a particular cause may be different from its corresponding effect on the probability of the event occurring. To overcome the related problems with interpretation with the CSHs approach, SDH that has a one-to-one correspondence with the cumulative incidence of the event was employed [19]. The SDH was modeled in a proportional hazard framework using the CIF.

To estimate the effect of covariates on the rate of occurrence of the outcome, the CSH and SDH regression models were used. In the presence of two possible types of failure, the relationship between cause-specific and sub-distribution hazards [21] were also derived in this study, and the partial likelihood techniques were employed to estimate the coefficients. The proportionality of hazards were also checked for CSH and SDH regression models, which is the main assumption required when modeling survival data. Further details on the methods employed for the statistical analysis are provided in the supplementary file (see Supplementary file 1). Descriptive statistics were used to describe frequency, percentage, and median of the study variables. Log rank and Gray’s test were used to estimate statistical significance for the categorical covariates. Once the data arrangement was accomplished all the statistical analyses were done using the *survival* and *cmprsk* packages of R statistical software (version 4.2.1).

## Results

### Demographic and clinical characteristics of TB patients

The data for this study consists of 457 patients who underwent TB treatments at AGH between January $1^{st}$, 2018, and January $30^{th}$, 2022. Of the 457 TB patients followed, 54 (11.8%) had LTFU TB treatment, 33 (7.2%) had died, and 370 (81%) had been censored, and these events occurred with median follow-up time of 4.2, 3.8, and 6 months, respectively. More than half (50.8%) of the study participants were females, and 17 (3.7%) and 15 (3.3%) of them were LTFU their treatment and died during the follow-up period, respectively. Comparatively, out of 225 (49.2%) male patients, 37 (8.1%) and 18 (3.9%) of them experienced LTFU and passed away, respectively. The majority of TB patients (88.4%) were new, while 21 (4.6%), 5 (1.1%), and 27 (5.9%) were relapsed, treatment failure, and transfer in patients, respectively. When observing the types of TB patients, 144 (31.5%) of them had extra pulmonary TB, 163 (35.7%) had smear positive pulmonary TB and 150 (32.8%) had smear negative pulmonary TB. Nearly one-fourth of the TB patients (24.4%) had lost their weight. Among the 103 (22.5%) HIV positive patients, 67 (14.7%), 23(5%), and 13 (2.8%) were censored, LTFU, and died, respectively. A contact history of TB illness was reported by more than half (57.3%) of the study subjects; of these, 217 (47.5%) were censored, 20 (4.4%) experienced LTFU, and 25 (5.4%) passed away. Additionally, less than 10% of TB patients were 18 years of age or younger, while more than 60% of patients fall into the 19 to 40 age group. Based on the baseline weight variable, nearly half of TB patients (48.1%) who had baseline weights between 38 and 54 kg were admitted to the hospital for treatment. The median follow-up period for all categories of the factors are also provided in Table 1.

**Table 1:** Descriptive summaries of demographic and clinical characteristics of tuberculosis patients

Variables	Categories	Censored	LTFU	Death	Med-	Total
Sex	Female	200(43.8%)	17(3.7%)	15(3.3%)	6.00	232(50.8%)
	Male	170(37.2%)	37(8.1%)	18(3.9%)	6.00	225(49.2%)
Residence	Urban	313(68.6%)	34(7.4%)	12(2.6%)	6.00	359(78.6%)
	Rural	57(12.4%)	20(4.4%)	21(4.6%)	6.00	98(21.4%)
Patient	New	335(73.3%)	44(9.6%)	25(5.5%)	6.00	404(88.4%)
category	Relapse	16(3.5%)	3(0.7%)	2(0.4%)	6.00	21(4.6%)
	Failure	3(0.7%)	2(0.4%)	0(0%)	5.27	5(1.1%)
	Transfer in	16 (3.5%)	5(1.1%)	6(1.3%)	5.57	27(5.9%)
Weight loss	No	295(64.6%)	29(6.3%)	22(4.8%)	6.00	346(75.7%)
	Yes	75(16.4%)	25(5.5%)	11(2.4%)	6.00	111(24.3%)
Types of TB	EPTB	125(27.4%)	8(1.7%)	11(2.4%)	6.00	144(31.5%)
	SPPTB	137(30%)	16(3.5%)	10(2.2%)	6.00	163(35.7%)
	SNPTB	108(23.6%)	30(6.6%)	12(12.6%)	6.00	150(32.8%)
HIV status	Negative	303(66.3%)	31(6.8%)	20(4.4%)	6.00	354(77.5%)
	Positive	67(14.7%)	23(5%)	13(2.8%)	6.00	103(22.5%)
Contact history	No	153(33.5%)	34(7.4%)	8(1.8%)	6.00	195 (42.7%)
	Yes	217(47.5%)	20(4.4%)	25(5.4%)	6.00	262(57.3%)
Age in years	$0 - 18$	38(8.3%)	5(1.1%)	0(0%)	6.00	43(9.4%)
	$19 - 40$	254(55.6%)	12(2.6%)	25(5.5%)	6.00	291(63.7%)
	$41 - 60$	52(11.4%)	15(3.3%)	8(1.7%)	6.00	75(16.4%)
	$>60$	26(5.7%)	22(4.8%)	0(0%)	5.75	48(10.5%)
Baseline	$<20$	10(2.2%)	14(3.1%)	0(0%)	5.73	24(5.3%)
weight in kg	$20 - 29$	27(5.9%)	11(2.4%)	0(0%)	6.00	38(8.3%)
	$30 - 37$	25(5.5%)	4(0.9%)	2(0.4%)	6.00	31(6.8%)
	$38 - 54$	183(40%)	17(3.7%)	20(4.4%)	6.00	220(48.1%)
	$\ge 55$	125(27.4%)	8(1.8%)	11(2.4%)	6.00	144(31.5%)
Total		370(81%)	54(11.8%)	33(7.2%)	-	457(100%)
Median		6.00	4.20	3.8	-	-

### Factors associated with LTFU TB treatment

The CIFs can be employed to statistically describe the survival data with competing risks for different causes of failures. In situations where competing risks are present, the KM approach may produce estimates that are biased because the competing risk event is treated as censored. As a result, the statistical significance of the categorical variables related to LTFU was first examined using the Chi-square (Log rank) test. The results are given in Table 2. The Chi-square analysis revealed that sex, place of residence, patient category, weight loss, types of TB, HIV status, contact history of patients, age, and baseline weight were significantly associated with LTFU (*p*-value < 0.05).

**Table 2:** Chi-square analyses (Gray’s and Log rank test) of factors related to TB patients with LTFU treatment

		Gray’s		Log	
Variables	Categories	test	*P*-value	rank test	*P*-value
Sex	Female				
	Male	8.84	0.003	9.6	0.002
Residence	Urban				
	Rural	8.73	0.003	13.5	$<0.001$
Patient	New				
category	Relapse	7.52	0.057	10	0.02
	Failure				
	Transfer in				
Weight loss	No				
	Yes	15.49	$<0.001$	17	$<0.001$
Types of TB	EPTB				
	SPPTB	15.50	$<0.001$	15.9	$<0.001$
	SNPTB				
HIV status	Negative				
	Positive	13.9	$<0.001$	16.1	$<0.001$
Contact history	No				
	Yes	10.1	0.002	9.4	0.002
Age in years	$0 - 18$				
	$$19 - 40$$				
	$41 - 60$	74.77	$<0.001$	79.4	$<0.001$
	$$>60$$				
Baseline	$<20$				
weight in kg	$20 - 29$				
	$30 - 37$	68.5	$<0.001$	76.5	$<0.001$
	$$38 - 54$$				
	$$\ge 55$$				

In addition, Gray’s test also called the modified chi-square test was used to assess the association between each potential prognostic factor and the outcome variable considered in the study, and the results are presented in Table 2. Since the CIFs for different causes of failure offer further insights into the survival data at hand, it is crucial to compare the cumulative incidence curves between different groups using the Gray’s test. The results based on the Gray’s test depicted difference between the cumulative incidence of groups such as sex, place of residence, weight loss, types of TB, HIV status, contact history of patients, age, and baseline weights with LTFU, while patient category is not.

**Fig. 1:**
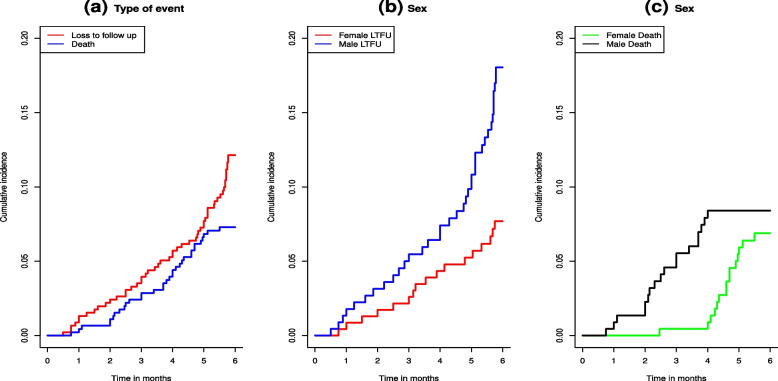
Cumulative incidence functions for LTFU and death (**a**), LTFU sex differences (**b**), and death incidences (**c**)

**Fig. 2:**
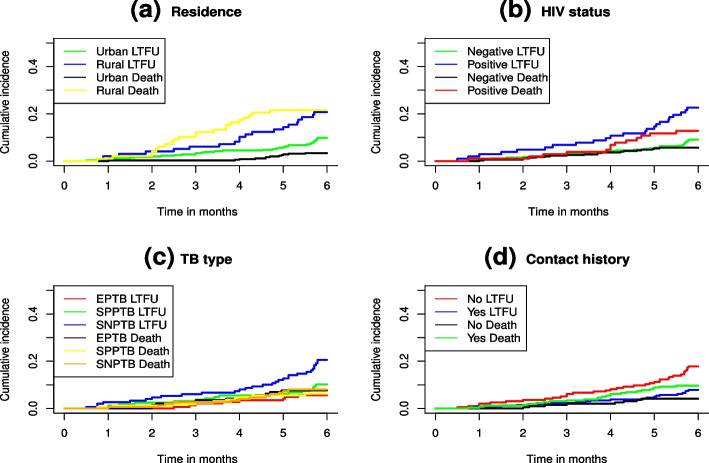
Non-parametric estimates of cumulative incidence curves with LTFU and death as a competing event for categories of residence in (**a**), HIV status in (**b**), TB type in (**c**) and contact history in (**d**)

Furthermore, non-parametric estimates of the CIFs for LTFU and death events are given in Fig. 1 for the outcome variables LTFU and death in (a), a comparison of male and female LTFU patients in (b), and a comparison of male and female death incidences in (c). From the plots in Fig. 1(a), the estimated probability of LTFU patients in the first six months was below 15%, while the estimated probability of dying in the first six months was below 8%. Considering the sex categories in Fig. 1(b), male patients had a higher estimated probability to experience LTFU in the first six months than female patients. Similarly, the estimated probability of dying in the first six months was higher for male than female patients (see Fig. 1(c)). Additionally, the non-parametric estimates of the cumulative incidence curves with LTFU, and death as competing events are given in Fig. 2. The plots can be used to compare the categories of selected variables, namely residence, HIV status, TB type, and contact history (Fig. 2).

### Estimation of the risk factors for LTFU

Before fitting the presented competing risk models to the dataset, the uni-variable analysis was employed to identify potentially significant covariates with 5% level of significance for inclusion in the multi-variable analysis. Based on uni-variable analysis, all potential covariates were incorporated in the multi-variable analysis of the competing-risk models since they were all significant at the 5% level of significance. As a result, we only present results from multi-variable analysis in the sections that follow, and variables with *p*-value < 0.05 were chosen as significant covariates.

#### Result for cause specific hazard regression

The assumption of proportional hazard (PH) was verified prior to analyzing each variable’s impact on the CSH adjusted for other factors. None of the covariates as well as global test were statistically significant (See Table 1 from Supplementary file). The plots of log (-log (S)) vs log (time) are also presented to check the assumption of cause-specific PH for the event of interest (LTFU) (See Supplementary file Fig. 1). The plots depicted no evidence against the assumption of proportionality for LTFU.

Then, the final model under the CSH using Cox regression model was fitted using multi-variable to estimate the effects of covariates on the CSHs for LTFU. The results are given in Table 3. According to the multi-variable CSH model, the CSHs for LTFU were significantly influenced by sex, residence, HIV status, contact history, age categories of 41-60, and 60 years and older, baseline weights of 38-54 kg, and above 55 kg. A male-to-female cause-specific hazard ratio (CSHR) of 2.336 (95% CI: 1.227 to 4.446) was found for LTFU. This indicates that male patients have a roughly 2.3 times higher risk of LTFU from TB treatment than female patients. Patients who lived in rural areas were more likely than those who lived in urban areas to face LTFU risk from TB treatment (CSHR, 3.092; 95% CI: 1.575 to 6.072).

**Table 3:** Multi-variable analysis of the cause-specific hazard regression model for LTFU

Variables	Categories	Estimate	SE	P-Value	CSHR (95% CI)
Sex	Female (Ref)				
	Male	0.849	0.328	0.009	2.336[1.227,4.446]
Residence	Urban (Ref)				
	Rural	1.129	0.344	0.001	3.092[1.575,6.071]
Weight loss	No (Ref)				
	Yes	0.241	0.311	0.439	1.272[0.691,2.341]
Types of TB	EPTB (Ref)				
	SPPTB	0.230	0.478	0.629	1.259[0.493,3.217]
	SNPTB	0.764	0.437	0.081	2.147[0.911,5.059]
HIV status	Negative (Ref)				
	Positive	1.131	0.302	$<0.001$	3.098[1.713,5.604]
Contact history	No (Ref)				
	Yes	-0.646	0.307	0.035	0.524[0.287,0.957]
Age in years	0-18 (Ref)				
	$19 - 40$	-0.031	0.603	0.959	0.969[0.297,3.161]
	$41 - 60$	1.554	0.538	0.004	4.729[1.649,13.565]
	$> 60$	2.146	0.549	$<0.001$	8.547[2.911,25.095]
Baseline	$<20$ (Ref)				
weight in kg	$20 - 29$	-0.284	0.462	0.538	0.7525[0.305,1.859]
	$30 - 37$	-1.149	0.665	0.084	0.317[0.086,1.167]
	$38 - 54$	-1.429	0.437	0.001	0.239[0.102,0.565]
	$\ge 55$	-2.029	0.499	$<0.001$	0.131[0.049,0.349]

Additionally, patients who were HIV positive had a roughly three-fold higher risk of LTFU from TB treatment than those who were HIV negative (CSHR, 3.098; 95% CI: 1.713, 5.604). TB patients who had a contact history had lower risks to experience treatment LTFU than patients who had no contact history (CSHR, 0.524). All other factors being held constant, the CSHR of LTFU is 4.729 and 8.547 times higher among the age groups 41-60 years and 60 years and older, respectively, than the age group 18 years and younger. When compared to baseline weights less than 20 kg, the CSHR of LTFU is 0.239 and 0.131 times lower in patients with baseline weights 38-54 kg and $\ge 55$ kg, respectively.

#### Result for sub-distribution hazard regression-fine and gray model

Similar to the CSH, an investigation in to the plot of log(-log(1-CIF)) vs log(time) was made to check the proportionality assumption of SDH for LTFU. The plot of log(-log(1-CIF)) vs log(time) revealed no evidence against the assumption of proportionality for LTFU (See Supplementary file Fig. 2). Then, the SDH regression model was fitted using multi-variable analysis and the covariates such as sex, residence, weight loss, types of TB, HIV status, contact history, age, baseline weights were found to be significant (Table 4). When considering death from any cause as a competing risk event, the categories of covariates such as sex, residence, HIV status, contact history, age categories 41-60, 60 years and older, baseline weights 38-54 kg, and above 55 kg were the major risk factors that led patients to LTFU treatment.

Specifically, when all other factors are held constant, the sub hazard ratio (SHR) of LTFU is 2.171 times greater in males than in females (95% CI: 1.196, 3.940). Patients whose place of residence was rural had a sub hazard of LTFU that is 2.716 times greater than patients whose place of residence was urban (95% CI: 1.444, 5.109). In patients with a contact history and HIV positive patients, the sub-hazard of LTFU is 3.044 and 0.490 times greater than in patients with no contact history and HIV negative patients, respectively (95% CI: 1.696, 5.462 for contact history; 0.263, 0.914 for HIV positive). In comparison to the age group of 18 years and younger, the risk of LTFU is higher for the age groups of 41 to 60 (SHR, 4.467) and 60 and older (SHR, 8.883).

**Table 4:** Multi-variable analysis of the sub-distribution hazard regression model for LTFU

Variables	Categories	Estimate	SE	P-value	SHR(95% CI)
Sex	Female (Ref)				
	Male	0.775	0.304	0.011	2.171[1.196,3.940]
Residence	Urban (Ref)				
	Rural	0.999	0.322	0.002	2.716[1.444,5.109]
Weight loss	No (Ref)				
	Yes	0.235	0.309	0.450	1.265[0.691,2.316]
Types of TB	EPTB (Ref)				
	SPPTB	0.175	0.469	0.710	1.191[0.475,2.986]
	SNPTB	0.718	0.410	0.080	2.050[0.918,4.577]
HIV status	Negative(Ref)				
	Positive)	1.113	0.298	$<0.001$	3.044[1.697,5.462]
Contact history	No (Ref)				
	Yes	-0.712	0.318	0.025	0.490[0.263,0.914]
Age in years	0-18 (Ref)				
	$19 - 40$	-0.088	0.734	0.900	0.916[ 0.217,3.857]
	$41 - 60$	1.497	0.589	0.011	4.467[1.409,14.158]
	$> 60$	2.184	0.633	$<0.001$	8.883[2.569,30.711]
Baseline	$<20$ (Ref)				
weight in kg	$20 - 29$	-0.233	0.431	0.590	0.792[0.341,1.843]
	$30 - 37$	-1.097	0.618	0.076	0.334[0.099,1.120]
	$38 - 54$	-1.465	0.540	0.006	0.231[0.080,0.666]
	$\ge 55$	-2.058	0.536	$<0.001$	0.128[0.045,0.365]

### Comparision of CSHR and SHR

When modeling time-to-event of interest in the presence of competing risks, it is recommended to report the outcome of both cause-specific Cox and Fine-Gray regression side by side [22, 23]. Based on the results obtained in the current study, the CSH ratio is greater than the SDH ratio for the covariates sex, residence, weight loss, SPPTB and SNPTB type of TB, HIV status, contact history, age categories 19-40 and 41-60 years old, and baseline weights 38-54 and $\ge$55 kg. However, CSH ratio is less than SDH ratio for the covariates age category 60 years and older and for the baseline weights 20-29 Kg and 30-37 Kg. This might be due to the fact that no death were reported for the age groups 18 years and younger as well as 60 years and older TB patients. Similarly, no deaths have occurred in the baseline weights below 29 Kg, while only 2 (0.4%) deaths occurred for the baseline weight 30-37 Kg (see Table 1). The maximum relative difference of the hazard ratios observed was 12.2%.

## Discussion

When analyzing time-to-event data with competing risks, various literatures point to the drawbacks or failures of classical time-to-event methods [20]. Most of these articles focused either on CSH or on SDH regression models (e.g., [24, 25]). In this study, regression models for the CSH and SDH were used to analyze time to LTFU, using death as a competing event in the data set. Moreover, the main ideas and theoretical background for these approaches were presented and compared regarding the intention of modeling, model assumptions and interpretation of the results obtained.

According to the analyses of the CSH and SDH models, the results obtained indicate that sex is significantly associated with time to LTFU treatment when death from any cause is considered as a competing risk event. The CSH and SDH for male patients were 2.3 and 2.1, respectively, suggesting that the CSH and sub-hazard of TB treatment LTFU for male patients are more than twice as high as female patients. This result is consistent with the study conducted by Enos et al. [26] in Kenya. Hence, it can be concluded that male patients were at higher risk of treatment interruption. The higher work rates and lower chance of seeking medical attention after the onset of suspected TB symptoms may explain this pattern, which has also been observed in TB research studied in other areas [27]. Similar studies conducted by Dangisso et al. [28] also showed a higher risk of LTFU in men.

Considering place of residence, patients in rural areas were over 2.7 times at greater risk of treatment LTFU than those in urban areas for the SHD model, but over 3 times at greater risk for the CSH model. This could be explained by the limited accessibility of treatment centers for patients residing in rural areas in Ethiopia. Otherwise, they must pay for public transportation to get to the treatment center, which may not always be an option because individuals in rural areas have lower income. This result is in agreement with the study conducted at JUSH, Jimma, Southwest Ethiopia [29] and other studies [30, 31]. The current study also revealed that the risk of LTFU TB treatment is over three times higher among patients who are HIV positive than HIV negative for both hazard models. Furthermore, the HIV non-reactive patients had a much lower risk of LTFU and improved survival time compared to those who were reactive to HIV, and the difference was statistically significant. This finding is consistent with the study by Shaweno et al. [31] and other similar studies [30]. Shaweno et al. [31] used the CPH model to assess factors associated with time to LTFU, and the time to LTFU of TB/HIV co-infected patients was nearly three-fold higher than those HIV sero-status negative.

The CSH and SDH in TB patients with a contact history were 0.524 and 0.490, respectively, times lower than those without a contact history. This is congruent with the findings of research undertaken by Baluku et al. [32] to determine if contact tracing is connected with treatment effectiveness in index TB patients in Uganda. Further, the CSH and SDH in patients aged 41-60 years were 4.729 and 4.467, respectively, indicating that the risk of LTFU in TB patients aged 41-60 years is more than four times higher than in those aged 18 years or younger. This result agrees with the study conducted by Abebe et al. [30] to assess treatment outcome and associated factors using logistic regression. Their study suggested that age was an independent risk factor for poor treatment outcome. Being aged 60 years and older is also associated with an increase in the rate of LTFU (CSHR 8.547 with 95% CI: 2.911, 25.095). These results are consistent with those of the cumulative incidence analysis and facilitate the interpretation of treatment effects on the cumulative incidence of LTFU. Indeed, higher LTFU rates in older patients were associated with lower rate of mortality in patients aged 60 years and older at the end of the study period. For example, Sheweno et al. [31] reported that the risk of LTFU from TB treatment increased by 70% with age, a finding consistent with other studies [33]. The findings of the current study also showed that weight was significantly associated with time to treatment LTFU in TB patients. For the baseline weights 38-54 Kg and $\ge 55$ Kg, the risk of treatment LTFU in TB patients was lower for both hazards compared to the baseline weight < 20 Kg. This result is supported by a study conducted at a public hospital in Harar Town, Eastern Ethiopia [34] and is consistent with other studies [35].

Overall, when comparing the hazard models, the current results revealed almost similar hazard ratios. However, higher covariate effects were observed when the CSH model was used for all covariates except for the age category 60 years and older and for the baseline weights between 20-29 KG and 30-37 Kg. For instance, Beyersmann et al. [21] used a real data example to compare competing risk analyses employing CSH and SDH models. When the proportionality assumption of the CSH model is met, Latouche et al. [36] investigated the results of the proportional SDH regression model. Their final conclusion was that effect estimates differed in both models, and the degree of difference depended on the cause-specific covariates effects on the event of interest and the effects on the competing event(s). Moreover, Dignam and Kocherginsky [37] extensively examined and reported the differences between the CSH and SDH regressions using several simulated situations. Their study demonstrated that the two groups comparison involving CSH or SDH ratios may differ significantly in the presence of competing events. These models emphasis on various measures, which may result in outcomes and interpretations that varied greatly. In order to prevent improper application of the methodologies and incorrect interpretation of the acquired data, researchers should be aware of the distinctions between CSH and SDH regression models.

Therefore, CSHR may be more appropriate if the focus is on investigating whether individuals belonging to one of the category of the factors are directly associated with LTFU. This measure was used at each time point to assess whether individuals in the category being compared had an increased instantaneous hazard rate of LTFU among all individuals surviving all events to this time point. Whereas, regardless of the direct association, whether the individuals in one of the categories were more likely to experience LTFU, the SDHR is a better measure of association. Under the SDH model, it remains possible to obtain a higher probability of LTFU among the categories if individuals were more likely to die prior to LTFU. Consequently, SDHR for LTFU would be higher for the category of the factor being compared, but this is achieved by reducing mortality and keeping individuals alive to experience LTFU. This is also the case for the baseline weight covariate in the current study.

## Conclusion

In this paper, the authors investigated the prognostic factors associated with time to LTFU of TB treatment at AGH using competing risk models. The death of TB patients from any cause was an event that competed with the event of interest, LTFU, in the study. This was addressed using a competing risk modeling approaches namely, CSH and SDH models. The assumptions of proportional hazards were checked first and then the partial likelihood technique was employed to estimate the parameters of the coefficients vectors. The results from applying both models revealed that sex, place of residence, HIV status, contact history, age, and baseline weights covariates were found to be statistically significant prognostic factors for time to LTFU TB treatment. The hazard ratios were also compared to identify the distinctions between the CSH and SDH regressions, and the effect estimates obtained from the hazard models were different.

The study recommends hospital authorities to pay attention on TB patients who are HIV positive, residing in rural areas, have no history of contact and are elderly. The rate of LTFU treatment was high for these groups. The proposed method will help physicians in clinical monitoring of TB patients and in assessing the efficacy of intervention packages. TB patients particularly those in the risk groups should be informed about the duration of DOTS and the consequences of interrupting treatment. Moreover, reducing the frequency of follow-up visits to receive medication and targeting high-risk groups in healthcare settings may help minimize the rates of LTFU in TB treatment. Further research should apply frailty model using competing risks with multiple endpoint to account for the effect of clusters and this is the subject of future research.

## Supplementary Information

**Additional file 1:** **Supplementary file.** Further details on the statistical methods, and results in the forms of Table and Figures used to check the assumptions of the CSH and SDH regressions are available here.

## Data Availability

The data sets analyzed in this study available from the corresponding author on reasonable request.

## Abbreviations

AGH: Ambo General Hospital
LTFU: loss-to-follow-up
TB: Tuberculosis
DOTS: Directly observed therapy short course
CIF: Cumulative Incidence Function
CSH: Cause Specific Hazard
SDH: Sub-distribution Hazard
CPH: Cox Proportional Hazard
EPTB: Extra pulmonary tuberculosis
SPPTB: Smear positive pulmonary tuberculosis
SNPTB: Smear negative pulmonary tuberculosis
