# Supplementary material for: Competing risk models to evaluate the factors for time to loss to follow-up among tuberculosis patients at Ambo General Hospital
Source: Arch Public Health. 2023 Jun 25;81:117. doi: 10.1186/s13690-023-01130-2 (PMC10290796; doi:10.1186/s13690-023-01130-2)
Supplement: Supplementary file 1 — Additional file 1: Supplementary file. Further details on the statistical methods, and results in the forms of Table and Figures used to check the assumptions of the CSH and SDH regressions are available here. [file 13690_2023_1130_MOESM1_ESM.pdf]

Supplementary file to: Competing risk models to evaluate the factors for time to loss to follow-up among tuberculosis patients at Ambo General Hospital

Authors: Daba Bulto Fufa, Tadele Akeba Diriba, Kenenisa Tadesse Dame and Legesse Kassa Debusho

Competing risk for analyzing survival data

Under the frame work of competing risks modeling the two most commonly used approaches are the CSH Cox approach and Fine-Gray proportional SDH model [1]. In the standard survival analysis, Cox proportional hazards model is a semi-parametric model in which dependence on the explanatory variables is modelled explicitly but no specific probability distribution is assumed for survival times. An analogous Cox regression approach can also be applied using CSHs regression when competing risks are present. In the analysis of times to a certain event k, the CSH is the instantaneous rate of experiencing cause k amongst those who are event-free (i.e., have not yet had cause k or any of the competing events). A straightforward way of applying this CSH approach is to fit a separate Cox model for each cause, censoring any competing events at their time of occurrence.

The one-to-one correspondence between hazard and survival that exists in the standard survival analysis does not necessarily hold when competing risks are present [2]. As a consequence, the effect of a covariate on the CSH for a particular cause may be different from its corresponding effect on the probability of the event occurring. To overcome the related problems with interpretation with the CSHs approach, Fine and Gray [1] introduced the concept of a SDH that has a one-to-one correspondence with the cumulative incidence of the event. The SDH can be modeled in a proportional hazard framework using the CIF. The CIF $F_k(t)$ estimates the probability of failing from cause k before a given time t to provide information for a certain population or to compare a discrete number of subgroups descriptively, and denoted by

$$F_k(t) = P(T \leq t, D = k), \tag{1}$$

with T and D being random variables representing the time to the first observed event and the type of event, respectively. Also, the CIF in a population or in subgroups of interest when competing risks are available can be estimated using

$$\hat{F}_k(t) = \sum_{i:t_i \leq t} \hat{\lambda}_k^{cs}(t)\hat{S}(t_{i-1}). \tag{2}$$

From expression (2), $\hat{S}(t)$ is the estimator for the overall survivor function at time t including all types of event and t_i denotes the ith ordered event time. $\hat{\lambda}_k^{cs}(t)$ is the CSH rate given by

$$\lambda_k^{cs}(t) = \lim_{\Delta t \to 0} \frac{P(t \leq T < t + \Delta t, D = k | T \geq t)}{\Delta t}, k = 1, 2, ..., D. \tag{3}$$

The CSH rate in expression (3) can be estimated by

$$\hat{\lambda}_k^{cs}(t) = \frac{d_{ki}}{n_i}, \tag{4}$$

where d_{ki} is the number of failures from type k at time t_i and n_i the risk set at time t_i, i.e. the number of patients who were not censored and have not failed from any cause up to time t_i.

Regression models for competing risks

Cause-specific Hazard Regression

The CSH regression model is used to estimate the effect of covariates on the rate of occurrence of the outcome in those subjects who are currently event free. If the covariate is continuous or the simultaneous effect of several covariates on cause-specific failure is of interest, a competing risks analogue of a CPHs model seems the most logical choice. The estimation of covariate effects on the CSH rate was proposed by Prentice et al. [3]. In a semi-parametric Cox regression approach [4] the CSH of cause k for subject with a covariate vector $\boldsymbol{X}$ is modeled using

$$\lambda_k^{cs}(t|\boldsymbol{X}) = \lambda_{k,0}^{cs}(t)\exp(\boldsymbol{\beta}_k^T\boldsymbol{X}), \tag{5}$$

where $\lambda_{k,0}^{cs}(t)$ is the baseline CSH of cause k and β_k is a vector of unknown parameters to be estimated for each outcome variable provided k is the cause of failure at time t.

The CSHs completely determine the competing risks process [5]. Hence, the CIFs can be estimated from separate CSH regression models for all types of event, for instance, the CIF for the kth out of $\boldsymbol{K}$ events is

$$F_k(t|\boldsymbol{X}) = \int_0^t \lambda_k^{cs}(s|\boldsymbol{X})\exp(-\sum_{l=1}^{k}\Lambda_l^{cs}(s|\boldsymbol{X}))ds, \tag{6}$$

where $\Lambda_k^{cs}(s|\boldsymbol{X})$ denotes the cumulative CSH rate for event k at time t for a given matrix of covariates $\boldsymbol{X}$ defined by

$$\Lambda_l^{cs}(s|\boldsymbol{X}) = \int_0^t \lambda_k^{cs}(s|\boldsymbol{X}).$$

Sub-distribution Hazard Regression-the Fine and Gray model

The Fine-Gray Model allows us to estimate the effect of covariates on the absolute risk of the outcome over time [1]. For event k, the SDH is the probability of a subject to fail from cause k in an inconsiderable small time interval Δt, given no event until time t or an event other than k occurred for a subject before time t [6],

$$\lambda_k^*(t) = \lim_{\Delta t\to 0}\frac{P(t \le T < t+\Delta t, D = k|T > t \cup (T \le t \cap D \ne k))}{\Delta t}. \tag{7}$$

Individuals failing before time t other than the cause of interest remain in the risk set for all future failure times. For events recorded in discrete time with no censoring, the SDH at time t_i can be estimated by

$$\hat{\lambda}_k^*(t_i) = \frac{d_{ki}}{n_i^*}, \tag{8}$$

where d_{ki} denotes the number of failures of type k at time t_i and n_i^* the modified risk set including all subjects who did not experience any event until time t_i and all subjects that failed before t_i from a cause other than k. The CIF is used to model the risk of experiencing a specific event in subjects who have not yet experienced this event. It denotes the instantaneous risk of failure from the kth event in subjects who have not yet experienced an event of type k. The basic difference between the two hazards is related to the risk sets. The risk set is the set of individuals/subjects under investigation and vulnerable to the event. In CIF, the risk set includes those who are currently alive as well as those who have previously experienced a competing event, while the risk set of the CSH function only considers those who are currently event free.

The SDH (hazard of the cumulative incidence) for each cause supposed as the hazard for an individual who either fails from cause k or does not can be written as

$$\lambda_k^*(t|\boldsymbol{X}) = \frac{f_k(t)}{1 - F_k(t)}. \tag{9}$$

Under the proportional hazard, Fine-Gray Model [7] can be specified as

$$\lambda_k^*(t|\boldsymbol{X}) = \lambda_{k,0}^*(t)\exp(\boldsymbol{\beta}_k^{*T}\boldsymbol{X}), \tag{10}$$

where $\lambda_{k,0}^*(t)$ denotes the baseline SDH for the cause of k. The SDH rates are assumed to be proportional for the included covariates and also directly linked to the CIF in a way known from the standard survival analysis with one possible endpoint given by

$$F_k(t|\boldsymbol{X}) = 1 - \exp(-\Lambda_k^*(t|\boldsymbol{X})) = 1 - exp(-\int_0^t \lambda_k^*(s|\boldsymbol{X})ds). \tag{11}$$

Hence, the CIF for the event of interest can be estimated directly from the regression coefficients obtained by a Fine and Gray model without explicit consideration of the covariate effects on competing events.

Model Comparison for the CSH and SDH regressions

In the presence of two possible types of failure, the relationship between cause-specific and sub-distribution hazards [5] can be derived using expressions (6) and (11) to get

$$\lambda_1^{cs}(t|\boldsymbol{X}) = (1 + \frac{F_2(t|\boldsymbol{X})}{S(t|\boldsymbol{X})}).\lambda_1^*(t|\boldsymbol{X}), \tag{12}$$

where $S(t|\boldsymbol{X})$ denotes the probability of being free of any event until time t given $\boldsymbol{X}$. Also, for each covariate the relative difference (RD) of the hazard ratios [8] can be computed by

$$\text{RD} = \frac{\text{SDHR} - \text{CSHR}}{\text{CSHR}} \times 100\% \tag{13}$$

Estimation of parameters

The partial likelihood are employed to estimate the coefficients vector [9]. Let $0 < t_{1k} < t_{2k} < ... < t_{uk}$ be ordered distinct time points at which failures of any causes occur for the risk k. Assume that only one failure can happen at each failure time, i.e. there are no tied failure times in the data. The partial likelihood [9] for specific hazard k is given by

$$L_k(\boldsymbol{\beta}_k) = \prod_{k=1}^{n_k} \frac{exp(\boldsymbol{\beta}_k^T \mathbf{X}_{ik}(t_{ik}))}{\sum_{l \in R(t_{ik})} exp(\boldsymbol{\beta}_k^T \mathbf{X}_{lk}(t_{lk}))}, \tag{14}$$

where n_k is the number of individuals in specific hazard k, $\mathbf{X}_{ik}$ is a vector of covariates for individual i specific to k-type risk at time t, the vector $\boldsymbol{\beta}_k$ represents the regression coefficients of cause k to be estimated. Since the same variables could have different effects on the different risks, $\boldsymbol{\beta}_k$ is independent of each other for each k and the set of individuals at risk at time t_{ik} is $R(t_{ik}) = \{l | t_{lk} \geq t_{ik}\}$. Using expression (14), the overall partial likelihood function is

$$L(\boldsymbol{\beta}) = \prod_{k=1}^{K} L_k(\boldsymbol{\beta}_k). \tag{15}$$

Estimation of parameters in the Fine-Gray model uses the partial likelihood approach by incorporating weights [10] given by

$$L(\boldsymbol{\beta}) = \prod_{i=1}^{n} \frac{exp(\boldsymbol{\beta}^T \mathbf{X}_i)}{\sum\limits_{l \in R(t_i)} w_{il} \; exp(\boldsymbol{\beta}^T \mathbf{X}_l)}, \tag{16}$$

where $\hat{\beta}_1 \;\hat{\beta}_2 \ldots \hat{\beta}_p$ are estimation of the regression parameters for event type k, $X_{i1} \; X_{i2} \ldots X_{ip}$ are predictor variables for subject i event type k. A collection of risks $R(t_i)$ defined by adding weights, which is based on non-censored subjects and can be concluded that collection of risks are formed based on the event of interest as $R(t_i) = \{l; T_l \geq t$ or $(T_l \leq t)$ and an individual in a competing risk$\}$.

Also, w_{il} in expression (16) is the weight of the subject i in the event of interest defined by

$$W_{ki} = \frac{\hat{G}(t_k)}{\hat{G}(min(t_i, t_k))}, \tag{17}$$

where $\hat{G}(t_i)$ is estimate of survival function from survival time i subject, $\hat{G}(min(t_i, t_l))$ is estimation of the survival function from the minimum value between the survival time of the subject i and the subject on the event l or event of interest. The weight will be 1 if the subject has a survival time $t_i \leq t_l$ and has a value ≤ 1 if $t_l > t_i$ [11]. Once the likelihood is formulated, the goal is to choose the values of parameters that maximize the likelihood.

Model Diagnostics

The main assumption when modeling survival data is the proportionality of hazards. When the Fine-Gray model is used, the hazards of the CIF must be proportional, whereas, in the CPH model, it is the CSHs that need to be proportional [11]. The proportionality assumption is the most common in competing risk regression models, which consider the sub-distribution with covariates $\mathbf{X}$ as a constant shift on the complementary log-log scale from a baseline sub-distribution function. If the curves do not cross with each other, then we say that the model does not violate the assumption of proportionality [12].

Once the data arrangement was accomplished all the statistical analysis was done using the *survival* and *cmprsk* packages of the R statistical software (version 4.2.1).

Result:Tables and Figures

Table 1 Test of proportionality assumptions of CSH model for LTFU

Variables	Chi-square	Dof	P-value
Sex	1.6196	1	0.20
Residence	1.2639	1	0.26
Patient category	2.5079	3	0.47
Weight loss	0.4959	1	0.48
Types of TB	1.2511	2	0.53
HIV status	0.0349	1	0.85
Contact history	0.2741	1	0.60
Age in years	3.1131	3	0.37
Baseline weight in kg	0.9545	4	0.92
GLOBAL TEST	10.6044	17	0.88

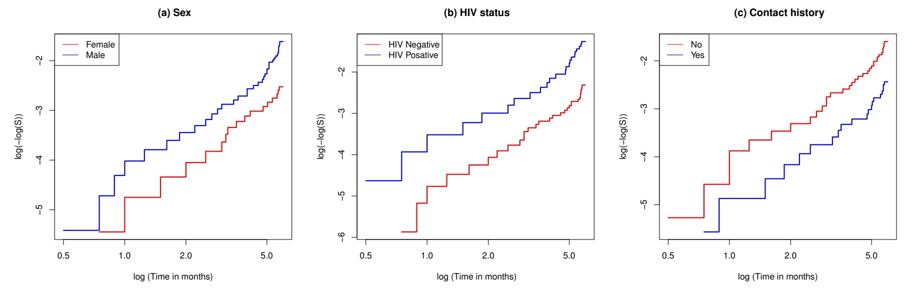

Figure 1 Cause-specific hazard proportionality for sex in (a), HIV in (b), and contact history in (c)

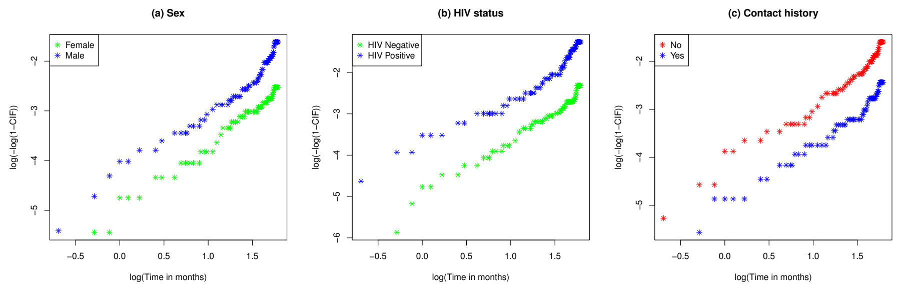

Figure 2 Plot of the proportionality for the hazard of CIF for sex in (a), HIV status in (b) and contact history in (c)

Author details

References

1. Fine JP, Gray RJ. A proportional hazards model for the subdistribution of a competing risk. Journal of the American statistical association. 1999;94(446):496–509.
2. Haller B, Schmidt G, Ulm K. Applying competing risks regression models: an overview. Lifetime Data Anal. 2013;19:33–58.
3. Prentice RL, Kalbfleisch JD, Peterson Jr AV, Flournoy N, Farewell VT, Breslow NE. The analysis of failure times in the presence of competing risks. Biometrics. 1978;p. 541–554.
4. Cox DR. Regression models and life-tables. Journal of the Royal Statistical Society: Series B (Methodological). 1972;34(2):187–202.
5. Beyersmann J, Latouche A, Buchholz A, Schumacher M. Simulating competing risks data in survival analysis. Statistics in Medicine. 2009;28(6):956–971168.
6. Gray RJ. A class of K-sample tests for comparing the cumulative incidence of a competing risk. The Annals of statistics. 1988;p. 1141–1154.
7. Leoce NM. Prognostic Modeling in the Presence of Competing Risks: An Application to Cardiovascular and Cancer Mortality in Breast Cancer Survivors. Columbia University; 2016.
8. Van Der Pas S, Nelissen R, Fiocco M. Different competing risks models for different questions may give similar results in arthroplasty registers in the presence of few events: Illustrated with 138,234 hip (124,560 patients) and 139,070 knee (125,213 patients) replacements from the Dutch Arthroplasty Register. Acta orthopaedica. 2018;89(2):145–151.
9. Cox DR. Partial likelihood. Biometrika. 1975;62(2):269–276.
10. Ainurrochmah A, Purnami S, et al. Survival analysis competing risk using fine-gray subdistribution model with the maximum partial likelihood estimation method. Materials Science and Engineering. 2021;1115:012–014.
11. Pintilie M. Competing risks: a practical perspective. John Wiley & Sons; 2006.
12. Zhang Z. Survival analysis in the presence of competing risks. Annals of translational medicine. 2017;5(3).
